# Comments on: “The Role of Muscle Mass Gain Following Protein Supplementation Plus Exercise Therapy in Older Adults with Sarcopenia and Frailty Risks: A Systematic Review and Meta-Regression Analysis of Randomized Trials”

**DOI:** 10.3390/nu11102406

**Published:** 2019-10-09

**Authors:** Wei-Ting Chen, Karen Chia-Wen Chu, Chyi-Huey Bai, Yuan-Pin Hsu

**Affiliations:** 1 Emergency Department, Wan Fang Hospital, Taipei Medical University, Taipei 116, Taiwan; 2 Department of Emergency, School of Medicine, College of Medicine, Taipei Medical University, Taipei 116, Taiwan; 3 Department of Public Health, School of Medicine, College of Medicine, Taipei Medical University, Taipei 116, Taiwan

We recently read with great interest the article titled “The Role of Muscle Mass Gain Following Protein Supplementation (PS) plus Exercise Therapy in Older Adults with Sarcopenia and Frailty Risks: A Systematic Review and Meta-Regression Analysis of Randomized Trials” [1]. After reading this article, we would like to address some issues.

First, the authors reported that PS plus muscle-strengthening exercise (MSE) significantly improved short-term and medium-term effects on lean body mass (LBM) and appendicular lean mass (ALM) [1]. However, the beneficial effect was noted in medium-term and long-term follow-up in Figure 2 [1]. 

Second, the authors found that PS + MSE provided different impacts on different participant types, participant conditions, and intervention periods for LBM [1]. They also stated that similar findings were observed for ALM. However, in Table 3, PS + MSE provided different effects on different qualities of their included study and participant conditions for ALM [1]. There are no differences in participant type and intervention periods. These findings need to be clarified.

Third, the authors intended to determine whether LBM gain was associated with muscle strength and physical mobility by using meta-regression analyses. In their Figures 3–5, they used dependent variables as the percent change in whole LBM or ALM [1], which was not clearly defined and may not be extracted in a *priori*. Besides, in Figure 3, a study produced a percentage change in LBM over 7, and in Figure 4, another study had a percentage change in ALM as low as −2, which are drastically different results from any other included studies [1]. These studies seem to be outliers. When these studies are excluded, the direction of the association may change significantly. Moreover, in multivariate meta-regression analyses, the authors adjusted for age, methodological quality, and follow-up time [1]. However, they did not adjust the critical variable of sex, which may have a different response to PS + MSE [2,3,4]. The proportion of sex varied substantially in the included studies, as showed in Table 1 [1]. Therefore, this variable should have been included in their meta-regression model. Furthermore, the 95% confidence intervals of the meta-regression model that they presented in Figures 3–5 were crossed [1], which makes for a confusing presentation. The authors need to clarify how these figures were produced and provide the regression equation and its R-square statistic.

Finally, they reported that PS + MSE provided better treatment effects on the handgrip, leg strength, and walking capability during an overall follow-up duration when compared to the control group [1]. However, as displayed in Figure 2, PS + MSE had a better beneficial response to handgrip, leg strength, and walking capability at medium-term follow up than control but not at long-term follow-up [1]. The authors should demonstrate this interesting finding.

Perhaps with these changes, this article can provide more accurate recommendations for protein supplements and exercise therapy in the elderly with sarcopenia and frailty risks.
